# Modeling psychiatric disorders using patient stem cell-derived neurons: a way forward

**DOI:** 10.1186/s13073-017-0512-3

**Published:** 2018-01-04

**Authors:** Krishna C. Vadodaria, Debha N. Amatya, Maria C. Marchetto, Fred H. Gage

**Affiliations:** Laboratory of Genetics, The Salk Institute for Biological Studies, 10010 North Torrey Pines Road, La Jolla, CA 92037 USA

## Abstract

Our understanding of the neurobiology of psychiatric disorders remains limited, and biomarker-based clinical management is yet to be developed. Induced pluripotent stem cell (iPSC) technology has revolutionized our capacity to generate patient-derived neurons to model psychiatric disorders. Here, we highlight advantages and caveats of iPSC disease modeling and outline strategies for addressing current challenges.

## The iPSC toolkit to capture genetic complexity

Even as neuropsychiatric research has boomed, psychiatric disorders have remained a leading cause of global morbidity and disease burden [1]. Currently, diagnosis is primarily qualitative, based on behavioral, cognitive, and emotional symptoms as delineated in the Diagnostic and Statistical Manual of Mental Disorders (DSM-5). The subjective nature of this existing clinical paradigm fails to incorporate biological data—for example, blood-based tests, imaging, or genetics—leading to unclear distinctions between disorders and hampering tailored therapeutic recommendations [2]. Research using animal models has provided insights into the neural mechanisms underlying endophenotypes, such as quantifiable components of the genes-to-behavior pathways associated with psychiatric disorders, but how precisely these findings can be extrapolated to human mental health remains difficult to assess [3]. Large-scale human genome-wide association studies (GWASs) for highly heritable polygenic psychiatric disorders, such as schizophrenia, have led to the discovery of numerous disease-associated but small effect-size variants. For less heritable and more heterogeneous disorders with a gradation of disease severity and variable sets of symptoms, disease genes are more difficult to identify, highlighting the need for patient-population stratification and larger sample sizes [4].

As a result, definitive diagnoses and treatment strategies based on objective biomarkers continue to evade us. The development of human iPSC technology offers one approach to allow researchers to address the genetic complexity issue in psychiatric disorder research. Somatic cells such as skin fibroblasts from adult patients can be dedifferentiated to a pluripotent state by transient overexpression of the reprogramming transcription factors. Theoretically, iPSC clones can then be differentiated to any other cell type by exposure to an appropriate combination of patterning molecules. Parallel in vitro disease modeling efforts for studying the neural correlates of disease-associated genotypes may provide novel insights into the neurological underpinnings of psychiatric disorders [5]. iPSCs and related transdifferentiation technologies have the capacity to generate previously inaccessible disease-relevant neural cell types from adult patients with known symptom histories, genetics, and drug response profiles. These cellular technologies facilitate the study of mental disorders at a scale and resolution previously not possible.

## Overcoming obstacles to neuropsychiatric disease modeling

A central goal of biological psychiatry is to understand how healthy and aberrant brain function may arise from the interaction of neural circuits. Crucial to this effort is generating relevant neural cell types from iPSCs, because studying the basic units of neural circuits in isolation enables the construction of in vitro model systems. Given the diversity of cell types in the mammalian brain, the field continues to develop protocols for generating relatively homogeneous populations of neuronal and glial subtypes, as well as genetic reporters to help label and identify specific cell types in mixed populations [6]. This approach is valuable for controlling cell-type heterogeneity so that appropriate comparisons can be made between individuals, which may enable the discovery of disease-associated cell type-specific defects and biomarkers. Given that specific neurotransmitter systems are targets of psychotropic drugs and have been implicated in the neuropathology of psychiatric disorders, another advantage of generating neurotransmitter- and region-specific neuronal subtypes is the ability to study pre- and post-synaptic cellular compartments in a segregated manner, which is difficult to do in vivo. Although it is possible to study brain regions in live patients with neuroimaging, or to use transcriptomic or histological analysis in post-mortem tissue, it is difficult to decipher from these methods whether observed differences are causative or a consequence of a lifetime of illness and pharmacological intervention. In vitro disease modeling offers an approach where such variables can be controlled for. Studies using iPSC-derived neurons from patients with psychiatric disorders such as schizophrenia, bipolar disorder, and autism spectrum disorders have uncovered disease- and, in some cases, gene-associated phenotypes in key processes such as progenitor cell proliferation, migration, neuronal morphology, connectivity, synaptic maturation, and neuronal activity [5]. For example, introducing a disease-associated mutation in the disrupted in schizophrenia 1 (*DISC1*) gene altered synaptic activity and downstream signaling in iPSC-derived neurons, establishing a causal relationship between patient genetics and cellular phenotypes [5]. Furthermore, drug treatment and transcriptome analyses from patient iPSC-derived cells have pointed to altered molecular signaling pathways as contributors to disease-associated in vitro cellular phenotypes [5].

## Fine-tuning the iPSC model system

**Fig. 1:**
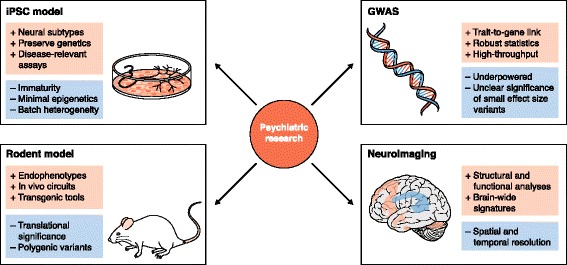
A summary of the strengths and weaknesses of common psychiatric research methods. The challenge of elucidating disease mechanisms in psychiatric disorders requires a diverse array of research tools. Each of these tools has unique strengths (*red*) and weaknesses (*blue*), thereby complementing one another. Here we summarize four techniques: induced pluripotent stem cell (*iPSC*) models, rodent models, genome-wide association studies (*GWAS*), and neuroimaging

While recent studies provide evidence for mechanisms that may contribute to disease pathology, excitement must be tempered by experimental knowledge addressing the caveats of in vitro disease modeling (Fig. 1). A downside of iPSC technology is a significant loss of epigenetic modifications after reprogramming, which poses a challenge for studying the impact of environmental factors on psychiatric disorders. However, it is possible that some epigenetic modifications are recapitulated following neuronal maturation in vitro [6]. Furthermore, iPSC-derived neurons are immature and their transcriptional profile is comparable to fetal neurons. Therefore, in vitro phenotypes may represent developmental phenomena preceding disease manifestation, presenting an opportunity for studying psychiatric disorders during development.

Another issue is that of variability between cell lines and across experimental batches, possibly due to somatic mosaicism in donor cells prior to reprogramming, accumulation of de novo mutations with selective advantages, stochastic events during differentiation, and heterogeneous patient genetics [6]. However, iPSC models which capture patient heterogeneity may provide a system for individually tailored assays for diagnostics and drug testing. As a complex picture of the variables at play emerges, complementary approaches and study designs addressing these caveats will be necessary to glean biologically meaningful information (Fig. 1).

One such approach is the stratification of large patient cohorts based on factors such as genetic risk, pharmacological response profiles, distinctive and quantitative endophenotypes, or comorbidities with other diseases. Modeling genetic risk includes rare variants conferring large genetic risk, such as copy number variation, or higher frequency common variants, such as single nucleotide polymorphisms, conferring relatively lower risk [4]. Cellular phenotypes associated with highly penetrant mutations can be studied using genome-edited isogenic iPSC lines or patient-derived iPSC lines. Experiments with the latter would ideally entail one-to-one comparisons between diseased and healthy individuals from the same family, controlling for genetic background. However, for idiopathic patient cohorts, genetic contributors are often unknown, and patient cohort segregation using drug responsiveness has proven to be a successful strategy for uncovering cellular phenotypes in, for example, schizophrenia and bipolar disorder [5]. Additionally, exploring the effects of pharmacological agents on human neural cells in vitro has indicated which molecular pathways and phenotypes may be therapeutically relevant. Collective data from such studies could provide deeper understanding of how diverse genetic risk factors converge on common biological processes and cellular phenotypes.

Another strategy is to study iPSC-derived neurons from a subset of well-characterized patients from a larger cohort. Here, in vitro phenotypes can be correlated with multiple continuous variables such as clinical severity, behavioral/biological measures, brain activity, and blood metabolites. Obtaining such multidimensional data from even small patient cohorts could inform the predictive value of individual variables and lead to the discovery of biomarkers. The explosion of rich neuropsychiatric disease sequence databases coincides with the emergence of powerful and accessible predictive machine learning tools. In conjunction with large-scale genetic data, deep-learning models may enjoy improved performance by using intermediate cellular phenotypes from patient-derived cells to bridge the gap between molecular and circuit or clinical features [7].

In addition to careful study design, picking appropriate in vitro models will be critical for discovering clinically relevant in vitro phenotypes. Three-dimensional iPSC-derived organoids may be able to recapitulate maturation-related signatures in developing circuits, as has been successfully done with autism spectrum disorder [8]. Similarly, transdifferentiation of adult somatic cells directly to neurons may partially conserve non-cell autonomous disease- and age-related epigenetic signatures that may be lost during reprogramming. Interestingly, processes such as inflammation have been implicated in psychiatric disorders, and microglia and astrocytes are emerging as central players in this process. Generating inflammation-sensitive glial cells from patient-derived iPSCs and co-culture experiments with neurons may prove useful for studying disease-relevant cellular interactions [9].

It is increasingly clear that gaining fresh insights into the biology of psychiatric disorders demands a multipronged approach, including but not limited to patient iPSC-based diseased modeling. Furthermore, concerted efforts across laboratories for tackling the inherent variability of in vitro systems may pave the way for establishing standardized in vitro parameters, which would be immensely helpful for moving toward high-throughput profiling and screening in the future [10]. Despite the gap in our knowledge of the biological causes underlying mental illness, iPSC technology—situated at the intersection of molecular biology and higher-order circuit properties—is well positioned to play an important role in disease study and biomarker discovery. We anticipate that, in the future, it may be possible to use patient iPSCs for predictive diagnoses and precision medicine.

## Abbreviations

DSM-5: Diagnostic and Statistical Manual of Mental Disorders
GWAS: Genome-wide associated study
iPSC: Induced pluripotent stem cell
